# Synthesis, molecular docking studies, and larvicidal activity evaluation of new fluorinated neonicotinoids against *Anopheles darlingi* larvae

**DOI:** 10.1371/journal.pone.0227811

**Published:** 2020-02-05

**Authors:** Rochelly da Silva Mesquita, Andrii Kyrylchuk, Iryna Grafova, Denys Kliukovskyi, Andriy Bezdudnyy, Alexander Rozhenko, Wanderli Pedro Tadei, Markku Leskelä, Andriy Grafov

**Affiliations:** 1 Department of Chemistry, Federal University of Amazonas, Manaus, Amazonas, Brazil; 2 Institute of Organic Chemistry, National Academy of Sciences of Ukraine, Kyiv, Ukraine; 3 Department of Chemistry, University of Helsinki, Helsinki, Finland; 4 Malaria and Dengue Laboratory, National Institute for Amazonian Research, Manaus, Amazonas, Brazil; Al-Azhar University, EGYPT

## Abstract

*Anopheles darlingi* is the main vector of malaria in Brazil, characterized by a high level of anthropophilia and endophagy. Imidacloprid, thiacloprid, and acetamiprid are the most widespread insecticides of the neonicotinoid group. However, they produce adverse effects on the non-target insects. Flupyradifurone has been marketed as an alternative to non-fluorinated neonicotinoids. Neonicotinoids containing trifluoroacethyl substituent reveal increased insecticidal activity due to higher hydrophobicity and metabolic stability.

We synthesized novel neonicotinoid insecticides containing fluorinated acceptor groups and their interactions were estimated with the nicotinic acetylcholine receptor (nAChR) binding site by molecular docking studies, to evaluate their larvicidal activity against *A*. *darlingi*, and to assess their outdoor photodegradation behavior. New neonicotinoid analogues were prepared and characterized by NMR and mass-spectrometry. The synthesized molecules were modelled by time-dependent density functional theory and analyzed, their interaction with nAChR was investigated by molecular docking. Their insecticide activity was tested on *Anopheles* larvae collected in suburban area of Manaus, Brazil. Four new fluorinated neonicotinoid analogs were prepared and tested against 3^rd^ instars larvae of *A*. *darlingi* showing high larvicidal activity. Docking studies reveal binding modes of the synthesized compounds and suggest that their insecticidal potency is governed by specific interactions with the receptor binding site and enhanced lipophilicity. 2-Chloro-5-(2-trifluoromethyl-pyrrolidin-1-ylmethyl)pyridine **5** showed fast degradation in water maintaining high larvicidal activity. All obtained substances possessed high larvicidal activity in low concentrations in 48 hours of exposure, compared to commercial flupyradifurone. Such activity is connected to a unique binding pattern of the synthesized compounds to insect’s nAChR and to their enhanced bioavailability owing to introduction of fluorinated amino-moieties. Therefore, the compounds in question have a high potential for application as control agents for insects transmitting tropical diseases, and they will be less persistent in the environment.

## Introduction

Malaria is a parasitic and endemic disease in several regions of the world with wide distribution mainly in tropical and subtropical areas. Currently, more than 3 billion people are at risk of contracting the disease and about 148 to 304 million of new cases are recorded annually [1].

*Anopheles darlingi* Root 1926 is the main vector of malaria in Brazil. On one hand, the species is characterized by a high level of anthropophily and endophagy in relation to other mosquito species of the Amazon region. On the other hand, *A*. *darlingi* is the malaria transmitting species the most favored by environmental changes resulting from human activities such as occupation of urban and peri-urban spaces in an uncontrolled manner; the construction of hydroelectric power plants, irrigation projects, and fishponds; the exploitation of fossil fuels, minerals, and natural gas; forest fires; deforestation; and road construction [2–5].

Mosquito vector control measures are the most commonly used approach to reduce the number of malaria cases. In relation to entomological data, the above measures include such traditional actions to combat vector-borne diseases as space spraying, indoor residual spraying, long-lasting insecticidal nets, larval source management, biolarvicide application, active search, and appropriate case treatment [6,7].

Management of larval breeding sites plays an important role in the control of malaria. Especially in those specific sites, where the vectors tend to reproduce, which may have characteristics of permanent or semi-permanent breeding sites. The WHO suggests the use of larvicides in those breeding sites located in urban areas, to protect the human population [5,8–10].

Larvicide control actions represent an effective tool for vector control, reducing the transmission of malaria in specific areas. This procedure is particularly important in the Amazonian region, where different breeding sites of malaria vectors are widely distributed [9].

According to the WHO recommendations, only the use of pyrethroid insecticides is indicated for the actions on adult mosquitoes in current vector control program [8,9]. Recently, resistance of the vector to these insecticides were reported in some areas, making research targeted at new classes of insecticides highly relevant [8,11].

The above dependence on the only one class of insecticides can be potentially overcome by implementation of neonicotinoids, a promising class of insecticides that exhibit excellent efficiency and low risk to humans and the environment. They are extensively used throughout the world for crop protection, particularly against sucking insect pests, accounting for one-fourth of the total world insecticide market [12]. Imidacloprid, thiacloprid, and acetamiprid are the most widespread insecticides of the neonicotinoid group. Toxicity of neonicotinoids to mammals is usually much lower than for many insect species. One can find reports on adverse effects of several widely adopted neonicotinoids on the non-target insects (especially honeybees) [13,14]. Prolonged periods of exposure of aquatic insects to neonicotinoids even though at low levels, has given rise to serious risks, since those insects are as sensitive to that class of insecticides, as the bees. Recently, imidacloprid toxic residues have been detected in water surfaces and are associated with the reduction of aquatic insect populations [15,16].

By that reason, the imidacloprid, thiamethoxam, and clothianidin were banned for use on flowering crops in the EU for the two-year term in 2013 [17,18]. After thorough risk assessment, the three pesticides were completely banned in the EU for outdoor use on crops since 2018 [19–21]. At the same time, it was established that several neonicotinoids (e.g. acetamiprid) represent low risk to honeybees [22,23], thus any further restrictions of those substances is neither scientifically nor legally appropriate [24]. Negative effects were mostly caused by improper and excessive use of the insecticides, or laboratory-based experiments that employed greater concentrations of the neonicotinoids than those found in nectar and pollen of the treated plants [17].

**Fig 1:**
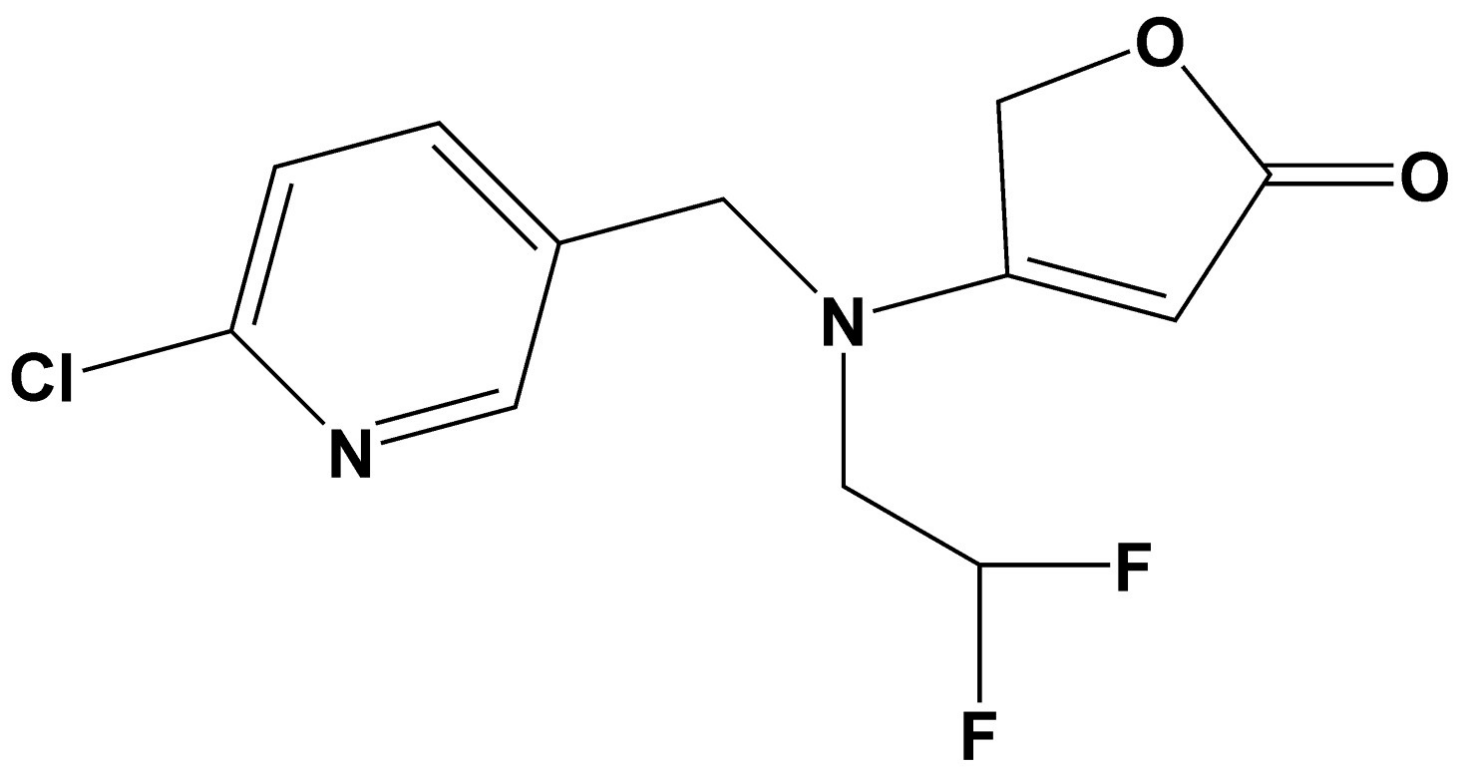
Flupyradifurone.

Flupyradifurone (**FPF**, Fig 1) is a new butenolide insecticide chemically similar to neonicotinoids, despite it has been marketed as an alternative to them for commercial reasons [25]. Both FPF and neonicotinoids have the same mode of action being a nicotinic acetylcholine receptor (nAChR) antagonists. The FPF possess low toxicity to non-target insects and mammals, and has recently been registered for use in Canada and the United States [26]. Like neonicotinoids, the FPF is highly water soluble and persistent in the environment [27]. However, it is practically non-toxic to young adult bees when in acute contact [28].

Agricultural and public health (vector control) importance of neonicotinoids have raised the need of their further development, taking into a consideration their mechanism of action. The nAChR is a biochemical target for neonicotinoids, it plays a central role in the mediation of fast excitatory synaptic transmission in the insect central nervous system [12]. Structural differences of nAChRs receptor subunits between vertebrate and in the invertebrate species [29] provides relatively low risk of neonicotinoids to mammals [29]. Interactions of the active compound with the nAChR may be efficiently investigated using the whole gamut of *in silico* methods, from inexpensive quantitative structure-activity relationships (QSAR) to computer docking and hybrid quantum and molecular mechanics (QM/MM) approaches [18,30–32].

In this study, we perform modeling of nAChR-ligand interactions via molecular docking. By analyzing various binding conformations and quick estimation of binding affinity for each of them, molecular docking allows finding of the best binding mode for virtually any potential ligand. This way we can rank compounds according to the strength of their interaction with the receptor and find specific intermolecular interactions that are important for the activity of a potential insecticide [33].

Trifluoromethyl and perfluoroalkyl groups can dramatically affect functional properties of organic molecules, thereby increasing a possibility of their application as agrochemicals, medicines or building blocks to create new organic materials [34–36].

Rationale for the introduction of fluorine atoms is based on the following reasons:

- Metabolic stability is one of the key factors in determining the bioavailability of a compound. A frequently employed strategy to circumvent this problem is to block the reactive site by a fluorine atom introduction. There are many examples illustrating an increase of a molecule metabolic stability by replacement of an oxidizable C-H group with a C-F one [37–40].
- Fluorine can change basicity of a compound. Presence of highly basic groups may limit bioavailability of a molecule. Introduction of the fluorine atom or fluorinated group in the vicinity of the basic moiety reduces the basicity. Thus, better membrane permeation and improved bioavailability may be expected for the fluorinated compound [41].
- Moreover, fluorine substituents are introduced to increase receptor-binding affinity of a compound. For example, most of the neurokinin1 receptor antagonist drugs contain a 3,5-di(trifluoromethyl)phenyl group to increase the binding affinity [42].

Earlier, it was shown that neonicotinoids containing trifluoroacethyl substituent reveal increased insecticidal activity due to higher hydrophobicity and metabolic stability [43–45]. Besides that, the presence of an acceptor substituent (such as a nitro- or a cyano-group) in a terminal moiety is one of the most important factors determining the insecticide activity [29].

Some pesticides have an obvious drawback, because of their possible persistence in the environment. In addition to being environmental contaminants, persistent insecticides develop a resistance to them much more frequently than non-persistent ones. Sunlight photodegradation is one of the most destructive pathways for pesticides after their release into the environment [46]. However, the biocidal activity should be preserved before the photodegradation occurs [47]. Several studies have been devoted to the investigation of the photolysis of the neonicotinoids [48–50]. Half-life of the FPF in water under sunlight was estimated to be as high as 2.5 days [51]. Therefore, reduced photostability becomes an important requirement in the design of new neonicotinoid insecticides.

During last few years we have developed synthetic approaches to generate novel low-molecular weight fluorinated amines and investigated their properties [52–54]. Substances of that kind have never been used as neonicotinoid building blocks. Therefore, the aim of this work was to synthesize new neonicotinoid insecticides containing fluorinated acceptor groups to estimate the interactions of the synthesized compounds with the nAChR binding site by molecular docking studies, to evaluate their larvicidal activity against *A*. *darlingi*, and to assess their outdoor photodegradation behavior.

## Materials and methods

3-trifluoromethylaniline was purchased from Acros Organics, all other reagents were purchased from Sigma-Aldrich and used as supplied. The solvents (Aldrich) were purified by standard procedures used in synthetic organic chemistry.

### Spectroscopy

^1^H NMR spectra were recorded on a Varian VXR-300 and Mercury 300 spectrometers, ^19^F NMR spectra were obtained on Gemini 200 Varian instrument at 188 MHz. UV- spectra were recorded on an UV-Vis Evolution 220 spectrophotometer (Thermo Fischer Scientific) in the range of 200–500 nm at a bandwidth of 2nm and integration time of 1s.

### General synthetic procedures

#### a) synthesis of fluorosubstituted anilines

3,5- Bis-trifluoromethylaniline was obtained following the two-step procedure with a total yield of 56% [55,56]. First, the 5-nitroisophthalic acid was fluorinated with SF_4_ taken in a 1:5 molar ratio at 80–901C for 16 hours in a presence of 10% of HF as a catalyst. The 3,5-bis(trifluoromethyl)nitrobenzene obtained was then reduced with hydrogen in a methanol solution over 10% Pd/C catalyst. The product was separated by distillation and identified spectroscopically. The spectra correspond to those described in [56].

4-(pentafluoroethoxy)aniline. 4-(pentafluoroethoxy)nitrobenzene was obtained according to the previously described procedure [57]. The fluorination product was then reduced with hydrogen in a methanol solution over 10% Pd/C catalyst, separated by distillation, and identified spectroscopically. The spectra correspond to those described in [58]. The total yield after two steps is 71%.

2-(trifluoromethyl)pyrrolidine was obtained according to our previously described procedure [52].

#### b) synthesis of neonicotinoids

**Fig 2:**
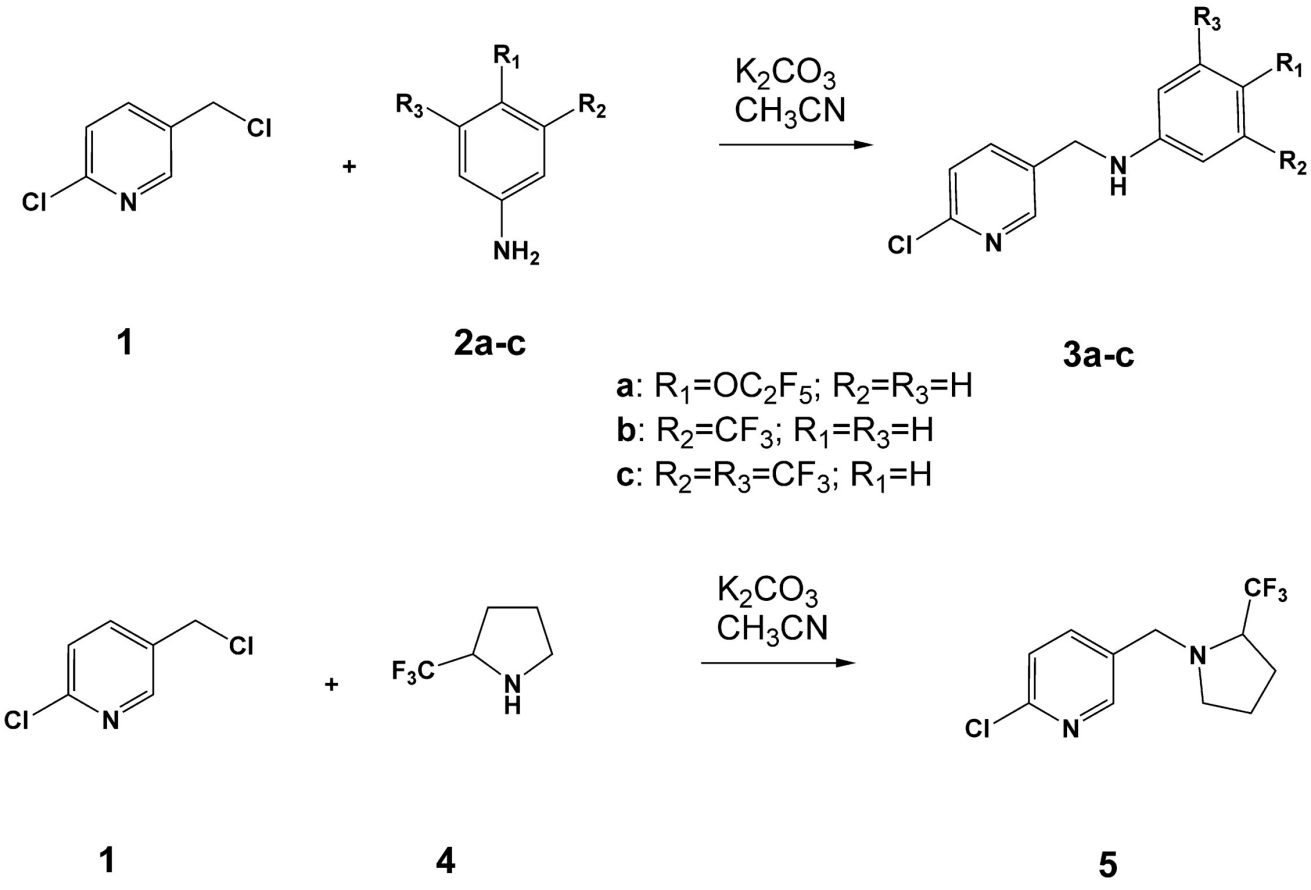
Synthesis of new neonicotinoids containing fluorinated acceptor groups.

New neonicotinoids were synthesized as shown in the Fig 2 according to the protocol http://dx.doi.org/10.17504/protocols.io.9h5h386.

A mixture of 2-chloro-5-(chloromethyl)pyridine **1** (0.324 g, 1.9 mmol), the corresponding substituted aniline **2a-c** or **4** (2.0 mmol), and anhydrous potassium carbonate (0.828 g, 6.0 mmol) in 5 ml of anhydrous acetonitrile was vigorously stirred under reflux (Fig 2). Progress of the reaction was monitored by thin layer chromatography (TLC). After the conversion was complete, the reaction mixture was separated from insoluble inorganic salts. The latter were thoroughly washed with dichloromethane. The combined organic solutions were evaporated under reduced pressure and the residue was purified by preparative thin layer chromatography (TLC) on SiO_2_ (EtOAc/hexane 1:2).

N-[(6-chloropyridin-3-yl)methyl]-4-(pentafluoroethoxy)aniline (**3a**). Yield 68%. Light-yellow viscous oil.^1^H NMR (300 MHz, CDCl_3_, TMS), *δ*: 3.93 (br. s, 1H, NH); 4.26 (s, 2H, CH_2_); 6.49 (d, 2H, C^3^’H, C^5^’H); 6.79 (d, 2H, C^2^’H, C^6^’H); 7.23 (d, 1H, C^3^H); 7.58 (m, 1H, C^2^H); 8.31 (s, 1H, C^5^H). ^19^F NMR (188 MHz, CDCl_3_, CCl_3_F), *δ*: -86.46 (s, 3F, CF_3_); -88.41 (s, 2F, CF_2_). ME (EI) m/z 353 (M+1, 100%).

N-[(6-chloropyridin-3-yl)methyl]-3-(trifluoromethyl)aniline (**3b**). Yield 61%. Light-yellow viscous oil.^1^H NMR (300 MHz, CDCl_3_, TMS), *δ*: 4.15 (br. s, 1H, NH); 4.29 (s, 2H, CH_2_); 6.64 (d, 1H, C^6^’H); 6.74 (s, 1H, C^5^’H); 6.89 (d, 1H, C^4^H); 7.16 (d, 1H, C^2^H); 7.22 (d, 1H, C^3^H); 7.57 (m, 1H, C^2^H); 8.23 (s, 1H, C^5^H). ^19^F NMR (188 MHz, CDCl_3_, CCl_3_F), *δ*: -62.09 (s). ME (EI) m/z 287 (M+1, 100%).

N-[(6-chloropyridin-3-yl)methyl]-3,5-bis(trifluoromethyl)aniline (**3c**). Yield 73%. Yellowish viscous oil. ^1^H NMR (300 MHz, CDCl_3_, TMS), *δ*: 3.59 (br. s, 1H, NH); 4.34 (s, 2H, CH_2_); 6.89 (s, 2H, C^2^’H, C^5^’H); 7.13 (s, 1H, C^4^H); 7.26 (d, 1H, C^3^H); 7.58 (m, 1H, C^2^H); 8.32 (s, 1H, C^5^H). ^19^F NMR (188 MHz, CDCl_3_, CCl_3_F), *δ*, m. d.: -62.55 (s). ME (EI) m/z 355 (M+1, 100%).

2-Chloro-5-(2-trifluoromethyl-pyrrolidin-1-ylmethyl)pyridine (**5**). Yield 83%. Light-yellow viscous oil. ^1^H NMR (300 MHz, CDCl_3_, TMS), *δ*: 1.82 (m, 2H, CH_2_C*H*_*2*_CH_2_ pyrrolidine), 2.01 (m, 2H, C*H*_*2*_CHCF_3_), 2.36 (m, 1H, CHCF_3_), 2.93 (m, 1H, CH*H*N pyrrolidine), 3.28 (m, 1H, C*H*HN pyrrolidine), 3.88 (ABX, ^2^*J*_HH_ = 153.4 Hz, ^4^*J*_HH_ = 13.6 Hz, 2H, ArCH_2_N), 7.29 (d, 1H, H-5 pyridine), 7.69 (dd, 1H, H-4 pyridine), 8.29 (d, 1H, H-3 pyridine). ^19^F NMR (188 MHz, CDCl_3_, CCl_3_F), -77.03 (s broad, 6F, C*F*_3_); ME (EI) m/z 265 (M+1, 100%).

### Calculation details

Electronic population analysis was conducted within the Gaussian-09 set of programs [59] using BP-86/TZVP approximation [60–62]. Multiwfn program was used for electrostatic potential (ESP) surfaces generation [63,64]. The TD-DFT calculations were carried out at the BP86/6-311G** approximation level using the Gaussian-03 program package. Molecular docking was carried out using AutoDock Vina package [65] and analyzed in AutoDockTools4 [66,67], VMD [68], and BIOVIA Discovery Studio 4.5 [69] programs were used for ESP surfaces and docking results visualization.

### Ethical considerations

The larvae used in this study were collected in suburban areas of the city of Manaus, Amazonas, Brazil. We declare that no permission was required for these locations. In addition, the collecting of anopheline larvae did not involve endangered or protected species.

### Test organism

*Anopheles* larvae were collected in suburban areas of the city of Manaus, Amazonas, Brazil and transported to insectary of the Laboratory of Malaria and Dengue at the National Institute of Amazonian Research (INPA). The mosquito larvae were reared according to the protocols described in [70]. Species of the larvae were identified after the realization of the bioassays.

All 7’920 larvae used in the bioassays were identified at the species level according to their morphological characteristics following the taxonomic keys of [71–73]. The specimens were placed in Petri dishes containing a few drops of larval fixative prepared according to the protocol adopted at the Laboratory of Malaria and Dengue of the INPA (http://dx.doi.org/10.17504/protocols.io.9qrh5v6) and observed under an optical microscope with magnifications of 100× to 400×. The identification of species was made based on the external morphological aspects, such as spiracular apparatus, head, antenna, apex, abdominal segments, palmate hairs, set of prothoracic hairs, thorax, dorsal view, and ventral integument appearance.

The colony was maintained at a temperature of 26 ± 2°C, a relative humidity around 70–80%, a photoperiod of 12:12 hours [70], and fed with a Tetramin^®^ fish food.

### Larvicidal activity

The larvicidal activity tests for new substances were performed on the 3^rd^ instar *Anopheles* larvae according to a methodology recommended by the WHO [74,75] https://dx.doi.org/10.17504/protocols.io.9kch4sw. All the experiments were carried out at the temperature of 26±2°C, the relative humidity of 70–80% and the photoperiod of 12:12 hours light/dark. For each substance, a stock solution in dimethylsufoxide (DMSO) was prepared with a concentration of 2000 μg/mL followed by a serial dilution to obtain 3.0, 5.0, 10.0, 20.0, and 30.0 μg/mL for **3a**; 2.5, 4.5, 9.5, 14.0, and 19.0 μg/mL for **3b**; 2.5, 4.5, 9.0, 14.0, and 18.0 μg/mL for **3c**; 6.0, 8.0, 10.0, 12.0, and 14.0 μg/mL for **5**; and 2.0, 3.0, 4.0, 5.0, and 6.0 μg/mL for **FPF**.

The experiment was performed in five replicates of each dosage and two controls: one of them containing 1.0% aqueous solution of DMSO (negative control) and the other one containing flupyradifurone (Sigma Aldrich^®^) as a positive control. Twenty larvae of the 3^rd^ instar were transferred into each dish of replicas and controls containing 50 mL of distilled water, food, and an aliquot of the stock solution in an appropriate amount for different concentrations. The larval mortalities were observed and registered at 48 hour intervals of exposure. Mortality rate of 10% and a confidence interval of 95% were set as the limits. The tests were repeated 3 times. The larvae were considered dead, when they were immobile and unable to reach the water surface.

### Statistical analysis

In order to obtain LC_50_, the mortality data were treated by a statistical software Polo plus [76], with 95% confidence interval and values of *p* < 0.05 were considered statistically significant. In the case of ≥10% mortality in the control group, the larval mortality was corrected using Abbott’s formula [77].

### Photodegradation test

40 mg/L solution of **5** was prepared using a deionized milli-Q water. The experiment was performed in Manaus (AM, Brazil; -3.096240ºS, -59.986194°W; under an average daily solar irradiation of 4.92 kWh/m^2^, http://www.cresesb.cepel.br/index.php#data). The test solution was placed into a quartz cuvette and tightly stopped there. The cuvette was places under a direct sunlight, maintaining the temperature at 35°C. Quantity of the compound **5** in the solution was monitored by UV-spectrophotometry, using Thermo Scientific Evolution 220- UV-vis spectrophotometer; Software INSIGHT: 1.3.10; Firmware 3.0.0.109; Scan Speed 120,00 nm/min; data Interval 1,00 nm; Integration Time 0,500 sec; Bandwidth 2 nm, following the absorption maximum at 268 nm. The spectra were recorded at different time intervals of 1h - 4h, and 1–4 days from 09:00 until 16:00 (i.e. 7 h each day) and analyzed according to [78].

## Results

**Fig 3:**
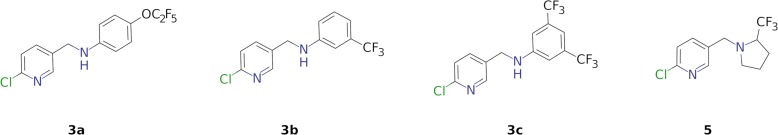
New neonicotinoids containing fluorinated acceptor groups.

New fluorinated neonicotinoids **3a-c** and **5** (Fig 3) were synthesized from 2-chloro-(5-chloromethyl)pyridine **1** and the corresponding fluorinated aromatic or alicyclic amines as shown in the Fig 2. The starting amines **2a-c** or **4** were refluxed with chloropyridine **1** in acetonitrile in the presence of potassium carbonate. Light-yellow viscous oils of the final compounds were obtained after their purification by preparative TLC.

**Fig 4:**
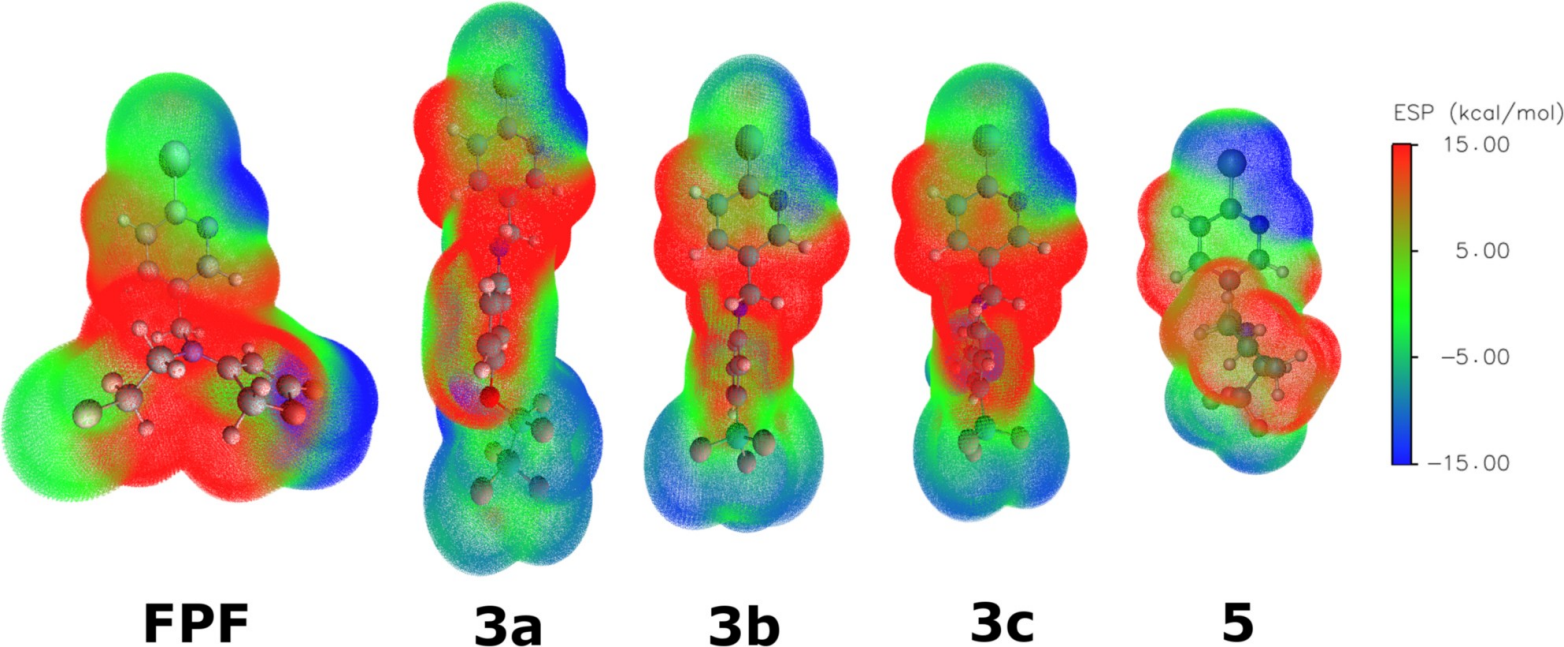
ESP surfaces for Flupyradifurone and new neonicotinoids 3a-c and 5.

In order to estimate structure-properties relationship for the substances in question, we performed DFT calculations of the molecules and generated their electrostatic potential surfaces (ESP) (Fig 4).

The ESP maps visualize electrostatic potential energy, i.e. energy of interaction between the molecule and an imaginary positively charged (+1) ion. Color scale in the right side of the figure shows a correspondence between the surface color and the interaction energy. Positive values (red) indicate higher interaction energy and therefore electron-poor positively charged part of the molecule. Negative values (blue) display opposite characteristics. Prevalence of the red and blue colors in the ESP maps evidence a polar character of the molecule, and appropriately colored regions point at regions of the structure that will more likely interact with positively (blue parts of the map) or negatively (red parts) charged groups of the receptor active site. Intermediate values of interaction energy (green) indicate weakly polar hydrophobic parts of the molecule.

**Fig 5:**
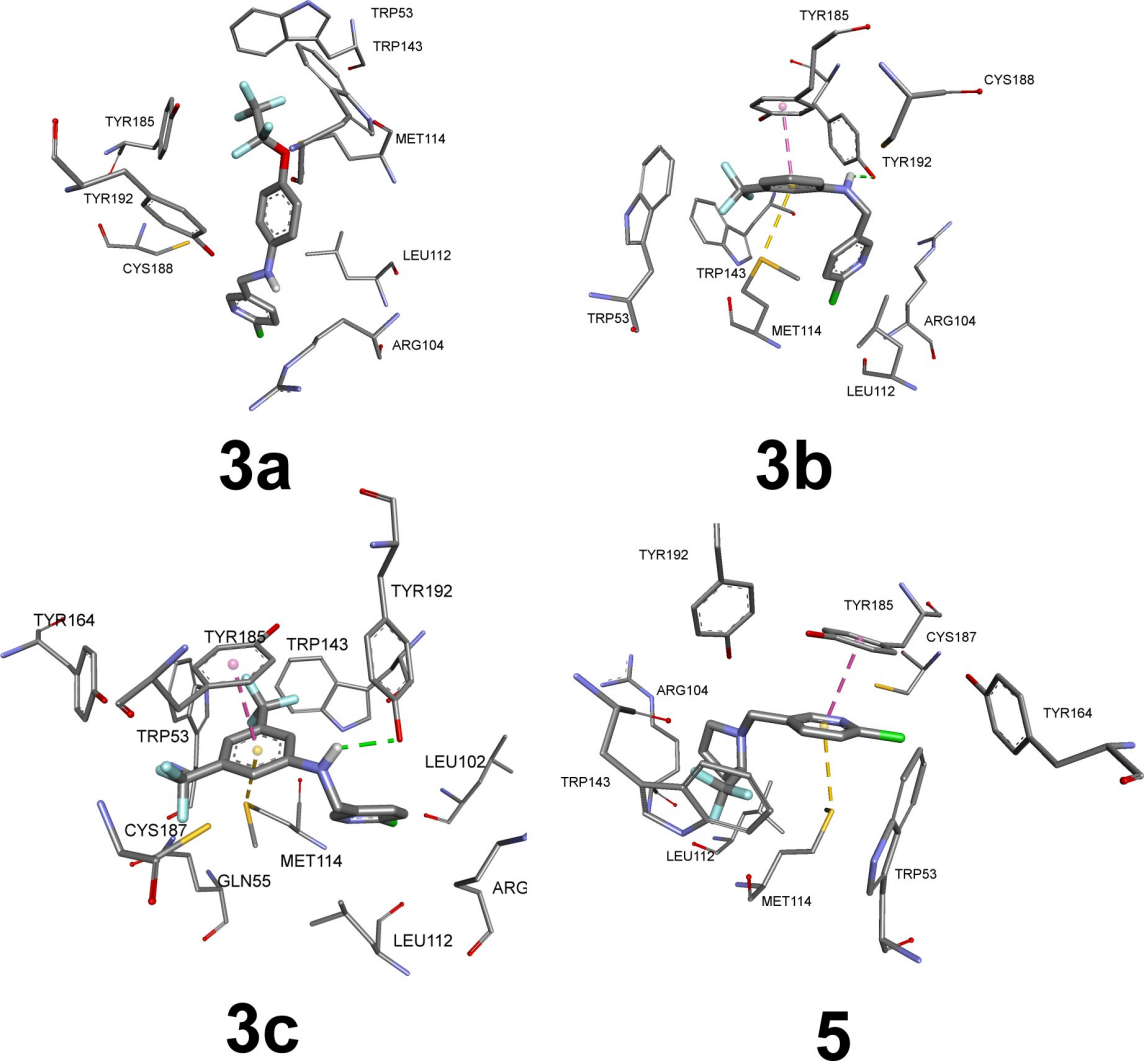
Binding modes of compounds 3a-c and 5 to *L*. *stagnalis* AChBP.

Possible binding models of the synthesized neonicotinoids with nAChR were examined by docking studies with AutoDock Vina [65] and shown in the Fig 5. Since it is known that amino acids forming binding pockets are conserved in an AChBPs (acetylcholine binding proteins), a crystal structure of a *Lymnaea stagnalis* AChBP co-crystallized with imidacloprid (PDB ID: 2zju) [79] was used as the receptor template.

The calculations also enabled to estimate the protein-ligand binding affinities and partition coefficients P shown in the Table 1.

**Table 1:** Estimated binding affinities and calculated logP values for the compounds 3a-c, 5, and FPF.

Compound	Predicted binding affinity, kJ/mol	logP
**3a**	-33.472	4.42
**3b**	-35.146	3.65
**3c**	-34.727	4.53
**5**	-30.125	2.94
**FPF**	-29.288	1.84

P = partition coefficient

Approximately 7’920 *Anopheles* larvae were collected in suburban areas of the city of Manaus (AM), Brazil. After the tests were performed successfully, all larvae were identified to verify the percentage of the species present. *A*. *darlingi* was the predominant species representing 77.38% of the larvae used in the tests. The remaining percentage was distributed among the following species: *A*. *nuneztovari* (16.36%), *A*. *triannulatus* (5.08%), *A*. *albitarsis* (0.80%), *A*. *oswaldoi* (0.20%), *A*. *evansae* (0.11%), *A*. *matogrossensis* (0.03%), and *A*. *nimbus* (0.01%).

The larvicidal activity of all synthesized compounds were evaluated, showing high mortality of the 3^rd^ instar larvae of *A*. *darlingi* (Table 2). The highest larvae mortality rates; i.e. the lowest LC_50_ values were observed for **3a**, **3c** and **3b** with the LC_50_ values of 0.57, 0.90 and 0.97 μg/mL respectively, which are lower than that of the commercially available FPF standard (LC_50_ = 1.18 μg/mL). Within the series under investigation, the highest LC_50_ value of 4.93 μg/mL was found for the compound **5**. No mortality was observed in the negative control.

**Table 2:** LC_50_ of new substances and FPF tested against 3^rd^ instars larvae of *A*. *darlingi* at the interval of 48 hours.

Substances	LC_50_ μg/mL	Regression equation	χ2	DF
**3a**	0.57	y = (0.26+5) + 1.10*log x	0.02	3
**3b**	0.97	y = (0.01+5) + 1.50*log x	4.70	3
**3c**	0.90	y = (0.06+5) + 1.35*log x	2.83	3
**5**	4.93	y = (7.33+5) - 5.08*log x	1.46	3
**FPF**	1.18	y = (3.02+5) - 0.22*log x	3.30	3

LC_50_: Median Lethal Concentration, χ2: Neill's lack-of-fit test, DF: degrees of freedom

**Fig 6:**
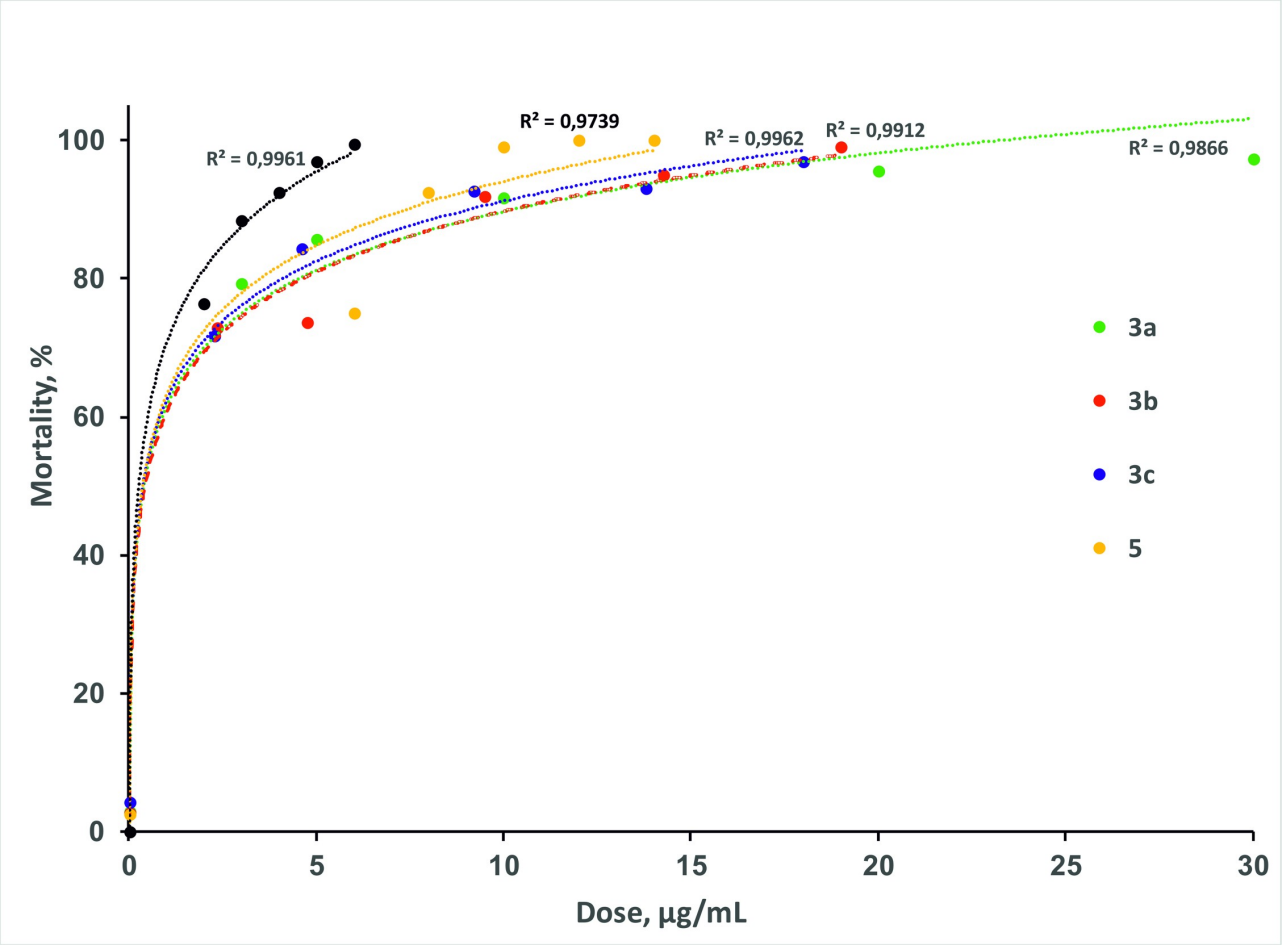
Mortality of *A*.*darlingi* larvae (%) *vs* concentration of the compounds 3a, 3b, 3c, 5, and flupyradifurone in the intervals of 48 hours. Trendlines are shown as the corresponding dash lines, R^2^ values are reported near each line.

The dose-response graphs (Fig 6) show scatter plots of the experimental data on percentage of mortality of *A*. *darlingi* larvae *vs* concentration and the trendlines for each of the compounds under investigation and the FPF. The coefficients of determination R^2^ show perfect fit of the data points by the regression trendlines. According to the graph, all tested compounds showed stable very high larvae mortality rates (more than 70%) starting from the lowest conscentrations applied. Thus, the data show high efficiency of the new substances against larvae of the main vector of malaria in the Amazonian region. The sample of commercial **FPF** insecticide was responsible for the highest larvae mortality. This implies that **FPF** was the compound showing the highest stability of action in the 48-hour interval, because it maintains its high larvicidal efficacy in the first doses tested (Fig 6). The compound **5** revealed a similar behavior, being the most efficient compound within the series under investigation. The compound **5** achieved 100% mortality rates at doses nearly 2 times lower than the compounds **3a-c**. By that reason, it was chosen for a photodegradation test under real outdoor conditions in the city of Manaus (AM, Brazil).

**Fig 7:**
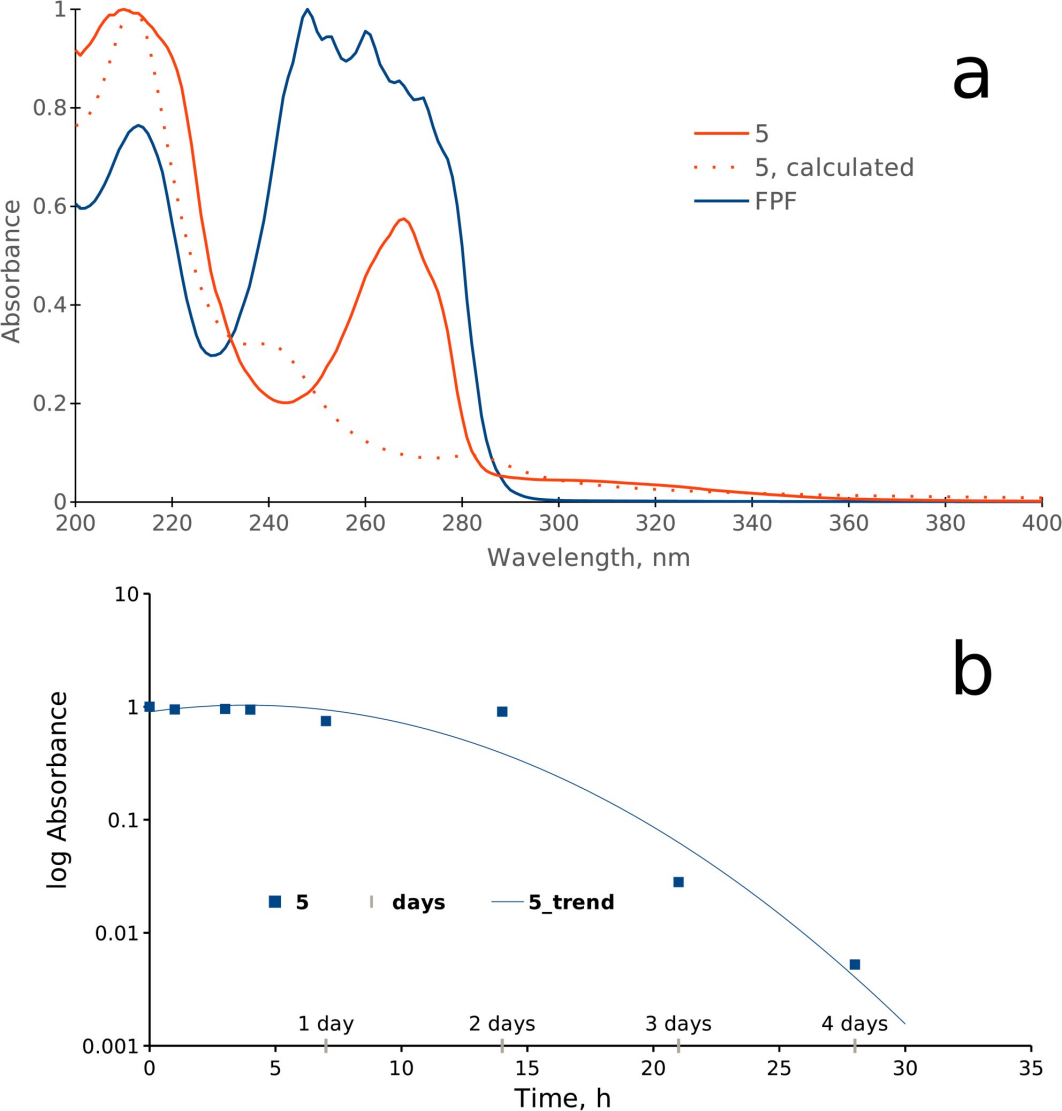
UV spectra. (a) spectra of the compound **5** and FPF. (b) Dependence of the absorption (at λmax = 268 nm) from the duration of irradiation.

The photodegradation was monitored by UV spectra. The UV spectra of **5** in aqueous solution showed absorption band maxima of 210 nm and 267 nm, which correspond well to the values predicted by TD-DFT calculations (Fig 7A). The outdoor photodegradation results are shown in the Fig 7B. As observed in the Fig 7, the compound starts to degrade shortly after 2 hours of exposure. The half-life of the compounds **5** was assessed from 0 h to 4 days of direct exposure to sunlight. The photodegradation half-life time for **5** (DT_50_ = 11.9 h) was derived based on UV/VIS spectral data according to the US Environmental Protection Agency standard operating procedure [78]. Comparison of residual deviances of the first- and second-order kinetic models shows that a single first-order rate model can be considered appropriate.

## Discussion

Various habitat forms, favorable environmental conditions for the development of mosquitoes, and their distribution in the environment, are the main factors that favor the diversity of species of the genus *Anopheles* in the Amazon region. The anophelines collected in the study region include *A*. *darlingi*, *A*. *nuneztovari*, *A*. *triannulatus*, *A*. *albitarsis*, *A*. *oswaldoi*, *A*. *evansae*, *A*. *matogrossensis*, and *A*. *nimbus*; all had previously been reported in the literature [2,3]. However, this diversity may vary according to the hydrological cycle, a phenomenon that influences the seasonality of species; also, a variety of different breeding sites, the intensification of human/vector contact, and the recorded cases of malaria have to be taken into account [5,80]. The dominance of certain anopheline species has also been related to their major ability to adapt to various environmental transformations, especially in the ecosystems altered by anthropic actions and environmental changes, such as increased exposure to sunlight and absence of frequently growing aquatic plants. The above factors affect the development of the mosquito larvae and cause a direct impact on the presence of different species of the same genus [80,81]. This study showed that *A*. *darlingi* was the dominant species in the collection area. It is considered the main malaria vector in the region, being more susceptible to infection by *Plasmodium spp*. parasites [80,81].

In the present study, we synthesized a series of new fluorinated neonicotinoid analogs starting from some novel amines containing -CF_3_ and -OC_2_F_5_ groups. Introduction of fluorine into a biologically active molecule increases its biological activity by affecting several parameters, e.g. binding to a target receptor. We suppose the presence of fluorinated amino-moieties will improve hydrophobic interactions with the receptor site.

Introduction of fluorine into a biologically active molecule increases its biological activity by affecting several parameters, e.g. binding to a target receptor. Thus, we synthesized a series of new fluorinated neonicotinoid analogs starting from some novel amines containing -CF_3_ and -OC_2_F_5_ groups. We suppose the presence of fluorinated amino-moieties will improve hydrophobic interactions with the receptor site.

In order to find structural features that may contribute to activity of the synthesized compounds compared to the FPF, the calculations of the electrostatic potential surfaces and molecular docking were carried out. Comparison of the molecular surface maps (Fig 4) shows a wide diversity of configurations for the compounds under investigation. The molecules of **3a-b** are bulkier than that of the FPF, a commercially available insecticide. Earlier, it was shown that bulky neonicotinoid analogs often have insecticidal potencies comparable to or even higher than popular commercial insecticides [31,43,82–84]. Such bulky substances can engage more regions of the ligand-binding domain of the nAChRs than conventional ones, or even dock to entirely different locations of those receptors [85,86].

Comparison of the ESP maps for the compounds **3a-c** and **5** with that of FPF (Fig 4) reveals two main distinctive features of the synthesized neonicotinoid analogues. First, the electronic density distribution and shape of the molecular surfaces vary substantially within the series and considerably differ from those of the FPF. Second, all the substances **3a-c** and **5** have enlarged weakly polar (green) lipophilic areas, capable to hydrophobic interactions with the receptor active site.

Binding modes of the synthesized neonicotinoids **3a-c** and **5** to nAChR are shown in the Fig 5. General feature of fluoroalkyl-phenyl substituted compounds **3a-c** is represented by orientation of the fluorinated groups and phenyl moiety to the hydrophobic region of the receptor binding pocket consisting of five aromatic amino acid residues–TRP53, TRP143, TYR164, TYR185 and TYR192. They contribute to the π-stacking interactions with phenyl rings at the distances of *ca*. 3.6 Å (that is within a usual range for parallel displaced π-stacking mode) [87] and to hydrophobic interactions with fluorinated groups. The stacking seems to have a large contribution to binding affinities, since the molecule of pyrrolydine derivative **5** is turned over, when compared to **3a-c** ones. Thus, the pyridine moiety can participate in stacking interaction with TYR185 phenyl ring. Three out of four compounds under discussion also take part in S-π interactions with MET114 sulfur atom at a distance of *ca*. 3.5 Å. The compound **3a** falls out of the row, evidently, because of the large volume of the -OC_2_F_5_ group. However, it is oriented similarly to the **3b** and **3c** allowing the hydrophobic interactions between the fluorine atoms of the neonicotinoid and aromatic amino acid residues.

Besides the non-covalent interactions described above, a hydrogen bonding between the NH-moiety of the neonicotinoid and a hydroxyl group of the TYR192 is present for **3c** and **3b** compounds. That bonding enhances stability of the receptor-ligand complex that is immediately seen in the results of molecular docking (compounds **3a,b** have the highest affinities). Measured larvicidal activities confirm the docking results, both compounds have low LC_50_ values.

According to the docking calculation results, the best predicted binding affinities do not vary significantly, being in the range of -30 ÷ -35 kJ/mol (Table 1). The LC_50_ values determined generally decrease along with a growing interaction strength. Fluorine substitution greatly enhances the lipophilicity of the amines employed for the synthesis. A common measure of lipophilicity of a compound is the partition coefficient P, *i*.*e*. ratio of its equilibrium concentrations in two immiscible liquids, usually octanol and water. Calculated logP values for the compounds under investigation and the FPF are shown in the Table 1. Comparison of the logP values for the compound **3b** having one CF_3_-group and the **3c** one with two CF_3_-groups shows that fluorine substitution has a substantial influence on the lipophilicity of the compound. Lethal concentrations of the substances **3a-c, 5** decrease with the increase of the calculated logP. The above results lead to an additional explanation of high larvicidal activity of the neonicotinoids in question. In addition to the binding strength to the insect’s nAChR, the enhanced activity of compounds **3a-c,** and **5** could also result from their high bioavailability due to facile blood-brain barrier crossing [29].

**Fig 8:**
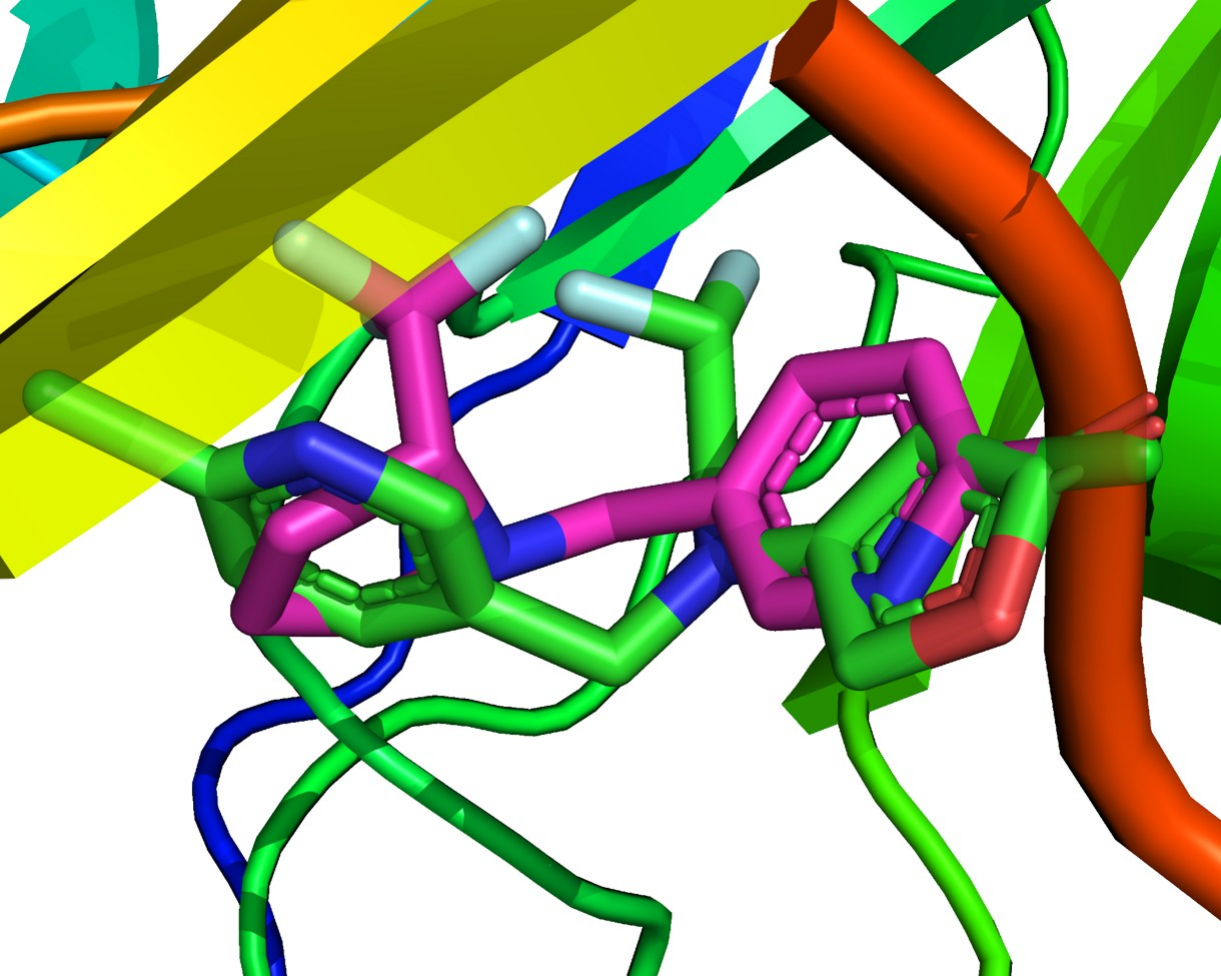
Comparison of the binding modes of FPF (green) and 5 (pink).

Position of the compound **5** at the receptor binding site is also very similar to that of flupyradifurone (Fig 8), which indicates similar receptor-ligand interactions of these compounds. CF_3_-group in **5** is a substitution of electronegative lactone pharmacophore of the FPF, while conformational mobility of **5** allows it to achieve simultaneous hydrogen bonding, aromatic interactions, and halogen bond.

Very high larvicidal activity of the new substances was confirmed in the test using *A*. *darlingi* larvae. The compound **3a** showed the best larvicidal potential (LC_50_ = 0.57 μg/mL) among the compounds under investigation, considering higher mortality of the larvae during the 48 h interval, even when compared to the FPF positive control LC_50_ = 1.18 μg/mL.

Since the compounds under investigation promoted a high mortality in contact with the larvae, they may be considered as potential larvicides. However, more specific tests are necessary to evaluate the mechanism of action and to classify them as possible insecticides to control the vectors of malaria in the Amazonian region.

Larvae of *Anopheles* mosquitos prefer to live in clean, unpolluted water. Despite a high insecticidal potential of neonicotinoids for vector control applications outdoors, quite a little is known about direct sunlight photodegradation in water of neonicotinoid insecticides containing fluorinated acceptor groups.

Since the habitat of *Anopheles* larvae is water, the eventual use of the compounds under real outdoor conditions may be modelled in an aqueous solution under intense natural sunlight and elevated environmental temperature, typical for the region. In this work we studied experimentally a photodegradation of **5** under real outdoor conditions (sunlight, environment, temperature) (Fig 7B) and compared those data to similar data for the FPF [25,88]. A reported DT_50_ value for the FPF (13.8 h) [88] is slightly higher than for the compound **5** (11.9 h). Hence, the 2-chloro-5-(2-trifluoromethyl-pyrrolidin-1-ylmethyl)pyridine would be less persistent in the environment.

Rather often, continuous and indiscriminate use of persistent in the environment insecticides in inadequate concentrations has led to the development of insect resistance to agents currently used in vector control. In the areas of Amazon region with high incidence of malaria, the application of insecticides is restricted to round the house places and some mosquito breeding site places. The former method is not very effective, since the houses are scattered in rural areas, and thus, the impact is minimal and should be monitored regularly. The latter is only permitted on a non-continuous base, when malaria outbreak happens and it is not possible to permanently eliminate the breeding site [9,89]. In such a way, environmental contamination may be avoided.

The fluorinated neonicotinoids under investigation showed high efficiency and rapid photodegradation; hence, they may be regarded as a viable option for insect vector control in places, where the resistance was observed. Another important safety factor is related to higher selectivity of neonicotinoids to the mosquito nAChRs [12,29] with respect to the mammalian ones.

The above would require further testing of the proposed compounds for their action on non-target organisms and persistence in the environment to ensure a more selective spectrum of action.

## Conclusion

Our results showed that the *A*. *darlingi* larvae were highly susceptible to new neonicotinoid analogs **3a-c** and **5** containing fluorinated acceptor groups. The substances **3a-c** revealed higher larvicidal activity at low concentrations in 48 hours of exposure, when compared to flupyradifurone. The reason of such activity may be connected to the unique binding pattern of the synthesized compounds to insect’s nAChR and to their enhanced bioavailability owing to introduction of fluorinated amino-moieties. The larvicidal activity tests showed the LC_50_ values that are in a good agreement with the theoretically predicted binding of the active compound to the nAChR. The photodegradation of **5** was studied under real outdoor conditions (sunlight, environment, temperature) and compared to the FPF data. The compound **5** degrades rapidly in water under direct sunlight, the DT_50_ value found was slightly lower than that of the FPF. Thus, in view of all the data reported in this study, the compound **5** has a lower persistence time in water without reducing its efficacy as a larvicide. However, further testing is required to evaluate those and other factors in real field conditions.

## Abbreviations

FPF: Flupyradifurone
nAChR: Nicotinic acetylcholine receptor
DT_50_: Degradation half-time
TD-DFT: Time dependent density functional theory
QSAR: quantitative structure-activity relationship
QM/MM: quantum mechanical molecular dynamics simulations
NMR: Nuclear magnetic resonance
TLC: thin layer chromatography
TMS: Tetramethyl silane
BP-86/TZVP: Functional composed of the Becke 1988 exchange functional and the Perdew 86 correlation functional / Triple zeta for valence electrons plus polarization function
DMSO: dimethylsufoxide
LC_50_: Median lethal dose
UV: ultraviolet
ESP: electrostatic potential surface
AChBP: acetylcholine binding protein
TRP: tryptophan
TYR: tyrosine
MET: methionine
